# Mortalidade por Doenças Cardiovasculares Segundo o Sistema de Informação sobre Mortalidade e as Estimativas do Estudo Carga Global de Doenças no Brasil, 2000-2017

**DOI:** 10.36660/abc.20190867

**Published:** 2020-08-19

**Authors:** Deborah Carvalho Malta, Renato Teixeira, Gláucia Maria Moraes de Oliveira, Antonio Luiz Pinho Ribeiro

**Affiliations:** 1 Universidade Federal de Minas Gerais Escola de Enfermagem Programa de Pós-Graduação em Saúde Pública Belo Horizonte MG Brasil Universidade Federal de Minas Gerais - Escola de Enfermagem, Programa de Pós-Graduação em Saúde Pública.,Belo Horizonte, MG - Brasil; 2 Universidade Federal de Minas Gerais Programa de Pós-Graduação em Saúde Pública Faculdade de Medicina Belo Horizonte MG Brasil Universidade Federal de Minas Gerais Programa de Pós-Graduação em Saúde Pública, Faculdade de Medicina,Belo Horizonte, MG - Brasil; 3 Universidade Federal do Rio de Janeiro Rio de Janeiro RJ Brasil Universidade Federal do Rio de Janeiro,Rio de Janeiro, RJ - Brasil; 4 Universidade Federal de Minas Gerais Departamento de Clínica Médica Faculdade de Medicina Belo Horizonte MG Brasil Universidade Federal de Minas Gerais - Centro de Telessaúde - Hospital das Clínicas/Departamento de Clínica Médica - Faculdade de Medicina, UFMG,Belo Horizonte, MG – Brasil

**Keywords:** Doenças Cardiovasculares/mortalidade, Sistema de Informação em Saúde/tendências, Confiabilidade dos Dados/tendências, Epidemiologia

## Abstract

**Fundamentos:**

O Sistema de Informação sobre Mortalidade (SIM) é de vital importância no monitoramento das tendências das doenças cardiovasculares (DCV), tendo por objetivo apoiar as políticas públicas.

**Objetivo:**

Comparar séries históricas de mortalidade por DCV tendo como fonte de dados o SIM com e sem correção e o estudo Carga Global de Doenças (GBD) 2017 no Brasil no período de 2000 a 2017.

**Métodos:**

Análise da mortalidade por DCV no Brasil entre 2000 e 2017 por meio de comparações de três estimativas de mortalidade por DCV: SIM Bruto, SIM Corrigido e GBD 2017. Foram utilizados os números absolutos e as taxas padronizadas por idade para comparação das estimativas para o Brasil e as unidades da federação.

**Resultados:**

No SIM, o total de óbitos por DCV variou de 261 mil, em 2000, a 359 mil, em 2017, e no GBD 2017, de 292 mil a 388 mil nos mesmos anos, respectivamente. Observou-se alta proporção de códigos *garbage* definidos pelo GBD 2017 nas causas de morte por DCV, chegando a 42% em 2017. As taxas de óbitos por 100 mil habitantes estimadas pelo GBD variaram de 248,8 (1990) a 178,0 (2017). As taxas do SIM Bruto e do SIM Corrigido também mostraram redução para toda a série analisada, sendo que o SIM Bruto apresentou taxas mais baixas, de 204,9 (1990) e 155,1 (2017) óbitos por 100 mil habitantes. Ao analisar por unidade da federação, as tendências do SIM Bruto se invertem, com aumento das taxas de mortalidade nos estados das regiões Norte e Nordeste.

**Conclusão:**

O estudo aponta a diminuição das taxas de mortalidade por DCV no período analisado para o Brasil. Ao contrário, na análise por unidade da federação, a variação porcentual do SIM Bruto foi de aumento para os estados do Norte e Nordeste. O uso dos dados não ajustados do SIM pode resultar em erros na interpretação, indicando aumento das taxas decorrente do aumento na captação de óbitos e da melhoria na definição das causas básicas de morte na última década, em especial nas regiões Norte e Nordeste, o que justifica sempre utilizar dados corrigidos na análise de mortalidade. (Arq Bras Cardiol. 2020; 115(2):152-160)

## Introdução

Nos últimos anos, o Brasil organizou diferentes fontes de dados que constituem os sistemas de informação de morbimortalidade e os inquéritos de saúde periódicos, que possibilitam monitorar de forma contínua mortalidade, morbidade e fatores de risco por doenças cardiovasculares (DCV), assim como obter informação para o processo de tomada de decisão em políticas de saúde.^1 , 2^

O Sistema de Informação sobre Mortalidade (SIM) foi implantado no Brasil em 1975, sendo o primeiro sistema de informação epidemiológica do Ministério da Saúde com abrangência nacional.^3^ O documento base do SIM é a declaração de óbito (DO), que deve ser preenchida por profissional médico. O médico que assistiu a pessoa que faleceu é o principal responsável pelo documento; na sua ausência ou falta, porém, a DO deve ser preenchida pelo médico substituto, pelo médico do Serviço de Verificação de Óbito – nos casos de morte por causa natural –, ou pelo médico legista do Instituto Médico Legal – para os óbitos por causas externas.^2 , 3^

O SIM constitui uma das principais ferramentas para o monitoramento das estatísticas de mortalidade no país, uma vez que todos os municípios do território nacional devem registrar seus óbitos, o que leva a cerca de 1,3 milhão de registros de óbito ao ano. Ocorreu aumento da cobertura do SIM em todas as unidades da federação (UF), passando de 86% em 2000 para 98% em 2017, embora alguns estados do Norte e Nordeste ainda mantenham coberturas menores que 95%.^1 - 3^ Houve ainda redução no número das causas mal definidas de óbito, cujas proporções, no entanto, ainda são elevadas em algumas UF. Em função disso, as análises de situação de saúde com base em dados de mortalidade devem ser realizadas com metodologias de correção capazes de minimizar o viés causado pelas causas mal definidas, pelos códigos *garbage* (CG) e pelo sub-registro de óbitos informados.^4 - 6^

Desde 1990, o estudo GBD ( *Global Burden of Disease* ) vem fazendo grandes avanços e uma mudança de paradigma na análise epidemiológica de dados secundários, ao propor um enfoque integrado das doenças e mortes, com a adoção de metodologia robusta e padronizada de análise, que contempla a correção dos CG, das causas mal definidas e do sub-registro.^7^ Os dados gerados pelo estudo GBD fornecem informações de 249 causas de morte para cerca de 195 locais, contemplando países e alguns níveis subnacionais, como para o Brasil e suas 27 UF. No estudo GBD, as informações sobre causas de morte foram coletadas de sistemas vitais de registro, sistemas de vigilância de mortalidade, pesquisas, registros hospitalares, registros policiais e autópsias verbais. No Brasil e em suas 27 UF, a fonte de dados de mortalidade é o SIM.^7 , 8^ No estudo GBD, diversos modelos estatísticos são utilizados com o objetivo de se obter a melhor estimativa para o número de óbitos para cada causa de morte segundo sexo e idade. O estudo GBD permite a comparação entre países, regiões e dados subnacionais, uma vez que faz uma padronização na qualidade dos dados de mortalidade dos locais. Além disso, o estudo GBD possibilita analisar as tendências populacionais, na medida em que os dados das séries temporais são ajustados e padronizados, possibilitando comparabilidade ao longo do tempo.^7 - 10^

Assim, o estudo atual visa comparar as séries históricas de mortalidade por DCV tendo como fonte os dados do SIM, com e sem correção, e do estudo GBD 2017 para o Brasil.

## Métodos

**Figura 1:**
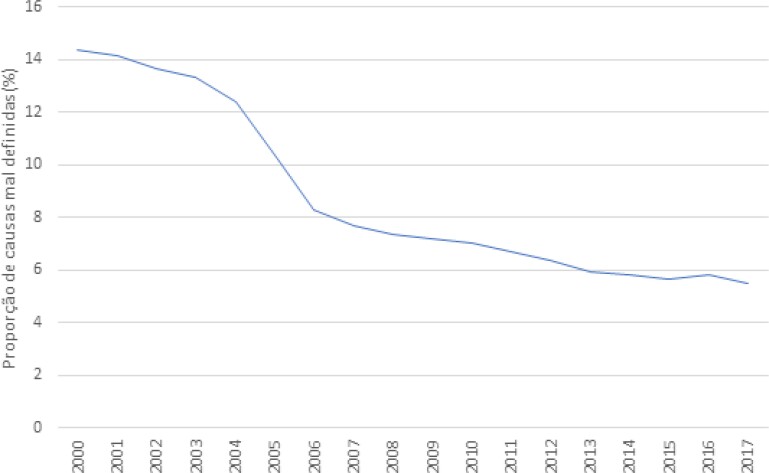
– *Proporção de causas mal definidas do Sistema de Informação de Mortalidade (SIM), Brasil, 2000 a 2017. Fonte: SIM.*

Realizou-se análise da série histórica da mortalidade por DCV no Brasil entre 2000 e 2017. A fonte de dados para o estudo foi o SIM, que contém as principais informações sobre os óbitos ocorridos em todo o país. Inicialmente foram descritas as proporções de causas mal definidas do SIM ( Figura 1 ).

Realizou-se a comparação de três estimativas de mortalidade por DCV: SIM Bruto, SIM Corrigido e GBD 2017. As estimativas do SIM, denominado no presente estudo SIM Corrigido e SIM Bruto, dependendo se houve ou não correção, tiveram como definição de DCV os códigos do capítulo IX (doenças do aparelho circulatório - I00-I99) da 10ª Classificação Estatística Internacional de Doenças e Problemas Relacionados à Saúde (CID-10). Já a classificação do GBD considera, inicialmente, os seguintes códigos: B33.2, G45-G46.8, I01-I01.9, I02.0, I05-I09.9, I11-I11.9, I20-I25.9, I28-I28.8, I30-I31.1, I31.8-I37.8, I38-I41.9, I42.1-I42.8, I43-I43.9, I47-I48.9, I51.0-I51.4, I60-I63.9, I65-I66.9, I67.0-I67.3, I67.5-I67.6, I68.0-I68.2, I69.0-I69.3, I70.2-I70.8, I71-I73.9, I77-I83.9, I86-I89.0, I89.9, I98 e K75.1.

**Figura 2:**
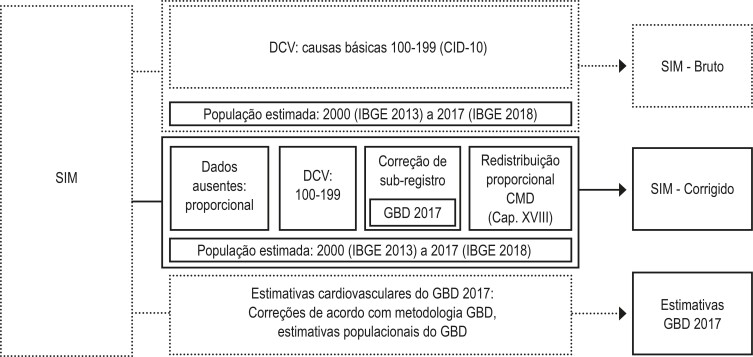
- Fluxograma das estimativas do Sistema de Informação de Mortalidade Bruto e Corrigido e do estudo Carga Global de Doenças, métodos de correção e população utilizada, para números e taxas de mortalidade. DCV – Doença Cardiovascular; IBGE – Instituto Brasileiro de Geografia e Estatística; SIM – Sistema de Informação sobre Mortalidade; GBD – Estudo Carga Global de Doenças; CMD – Causas Mal Definidas.

A Figura 2 mostra os métodos de correção de óbitos e população utilizados para estimar os números absolutos e taxas de mortalidade para o SIM Bruto e o SIM Corrigido e as estimativas do estudo GBD 2017. O numerador consiste nas DCV (I00-I99) registradas pelo SIM. As estimativas geradas pelo SIM Bruto foram padronizadas por idade, não tendo sido aplicadas outras correções. As estimativas geradas pelo SIM Corrigido foram padronizadas por idade e corrigidas: quanto ao sub-registro, utilizando-se a metodologia do GBD; quanto aos óbitos sem informação de idade e sexo, utilizando-se a redistribuição proporcional desses óbitos; e quanto às causas mal definidas, utilizando-se a redistribuição proporcional dessas causas entre os grupos de causas cardiovasculares e os demais capítulos.^4^ As estimativas do GBD 2017 foram extraídas da base de dados do *Institute for Health Metrics and Evaluation* (IHME) e submetidas às correções previamente descritas e detalhadas em publicações anteriores, entre as quais as correções para sub-registro, para CG e para causas mal definidas.^7 , 8^

Os dados do SIM, brutos e corrigidos, foram comparados com as estimativas geradas pelo estudo GBD, que também tem como fonte os dados do SIM, tanto pela série histórica no período 2000 a 2017, segundo os óbitos totais e em números absolutos do capítulo de DCV *,* quanto pelas taxas padronizadas por idade das três estimativas. Tanto no SIM Bruto quanto no SIM Corrigido, as taxas foram calculadas tendo como denominador as estimativas populacionais mais atualizadas geradas pelo Instituto Brasileiro de Geografia e Estatística (IBGE).^11^ Porém, como essa estimativa do IBGE só apresenta dados a partir de 2010, aplicaram-se duas interpolações: uma com os dados do ano de 2000 da versão 2013 disponibilizada pelo IBGE^12^ e outra com os dados do ano de 2010 da atual versão disponibilizada em 2018.^11^ A população padrão utilizada para o ajuste das taxas padronizadas por idade, por uso do método direto, foi a população mundial do estudo GBD.^7^ Para o cálculo das taxas, o estudo GBD considera suas próprias estimativas populacionais (GBD Fonte). Todas as três estimativas foram analisadas para os códigos do conjunto das causas de morte cardiovasculares (I00-I99) nos anos de 2000 a 2017. A Figura 2 apresenta o fluxograma utilizado para comparação das três estimativas, segundo os números absolutos de óbitos e as taxas de mortalidade para o Brasil e as UF.

As análises foram realizadas empregando-se o *Stata Statistical Software,* versão 14 *(College Station, TX: StataCorp LP)* .

## Resultados

A Figura 1 mostra a proporção das causas mal definidas em relação ao total de óbitos no Brasil de 2000 a 2017. Essa proporção foi de 14,3% em 2000, observando-se uma diminuição mais acentuada a partir de 2005, atingindo 5,5% em 2017.

A Tabela 1 mostra que grande parte dos óbitos com causas básicas definidas pelos códigos do capítulo IX da CID-10 constitui CG, usando-se a definição de CG do estudo GBD 2017, sendo a proporção de CG de 42,1% em 2017. Observa-se ainda que, com o passar dos anos, o número de CG diminuiu lentamente, indicando uma melhora na qualidade das definições das causas de morte do capítulo IX da CID-10. O estudo GBD redistribui os CG nas suas estimativas.

**Tabela 1:** – Número total de óbitos, números absolutos e porcentagens de óbitos por doenças cardiovasculares de acordo com o capítulo IX da CID-10 (I00-I99) e com as definições do GBD para doenças cardiovasculares, e ainda números absolutos e porcentagens de códigos *garbage* , no Brasil, de 2000 a 2017.

Ano	Total	I00-I99		Cardiovascular GBD		Códigos *garbage*	

		n	%	n	%	n	%
2000	946.685	260.603	27,5	129.883	49,8	126.803	48,7
2001	961.492	263.417	27,4	130.774	49,6	128.637	48,8
2002	982.807	267.496	27,2	133.160	49,8	130.362	48,7
2003	1.002.340	274.068	27,3	137.413	50,1	132.245	48,3
2004	1.024.073	285.543	27,9	143.811	50,4	137.096	48,0
2005	1.006.827	283.927	28,2	142.656	50,2	136.545	48,1
2006	1.031.691	302.817	29,4	152.017	50,2	145.977	48,2
2007	1.047.824	308.466	29,4	156.253	50,7	147.076	47,7
2008	1.077.007	317.797	29,5	163.255	51,4	149.058	46,9
2009	1.103.088	320.074	29,0	164.036	51,2	150.082	46,9
2010	1.136.947	326.371	28,7	167.974	51,5	152.326	46,7
2011	1.170.498	335.213	28,6	173.397	51,7	155.363	46,3
2012	1.181.166	333.295	28,2	174.750	52,4	152.276	45,7
2013	1.210.474	339.672	28,1	179.200	52,8	153.822	45,3
2014	1.227.039	340.284	27,7	181.223	53,3	152.421	44,8
2015	1.264.175	349.642	27,7	186.570	53,4	156.278	44,7
2016	1.309.774	362.091	27,6	194.987	53,9	159.779	44,1
2017	1.312.664	358.882	27,3	199.872	55,7	150.967	42,1

*Óbitos do capítulo de cardiovasculares não classificados como doenças cardiovasculares e códigos garbage não apresentados na tabela, média de 1,8%.

**Figura 3:**
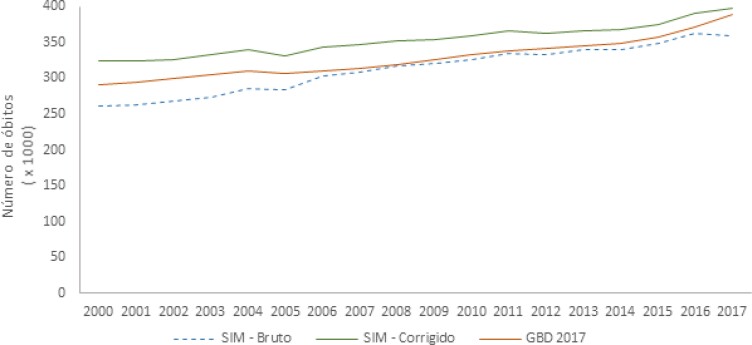
– *Números absolutos de óbitos por doenças cardiovasculares no SIM, bruto e corrigido, e no GBD 2017. Brasil, 2000 a 2017. Fonte: SIM e GBD 2017*

Foram analisados os números absolutos de óbitos e as taxas de mortalidade padronizadas para o SIM, Bruto e Corrigido, e para as estimativas do GBD 2017. A Figura 3 mostra os óbitos para as três estimativas em números absolutos, com aumento similar para todos os três métodos utilizados. Foram registrados aproximadamente 261 mil óbitos em 2000 no SIM, chegando a 359 mil óbitos no ano de 2017. Após as correções, os óbitos registrados no SIM Corrigido foram 324 mil e 397 mil, em 2000 e 2017, respectivamente. As estimativas GBD apresentaram crescimento de 292 mil óbitos para 388 mil óbitos nos mesmos anos analisados.

**Figura 4:**
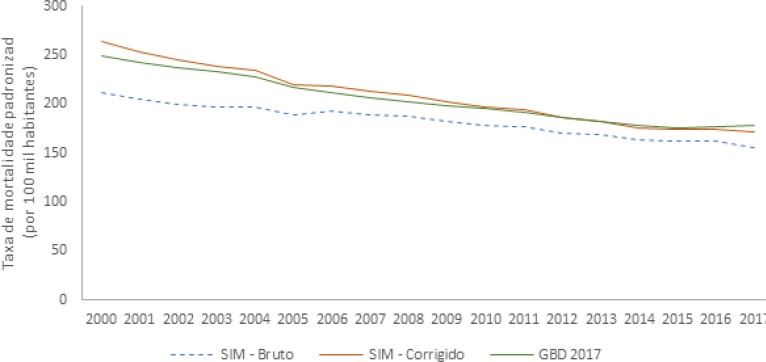
– *Taxa de mortalidade por doenças cardiovasculares padronizada para o SIM, bruto e corrigido, e para o GBD 2017. Brasil, 2000 a 2017. Fonte: SIM e GBD 2017*

As taxas de mortalidade por DCV mostraram redução no período analisado ( Figura 4 ). As do SIM Bruto variaram de 211,7 a 155,1 óbitos por 100 mil habitantes, enquanto, após a correção, as do SIM Corrigido foram de 263,9 a 172,0 óbitos por 100 mil habitantes. As taxas de mortalidade por DCV estimadas pelo GBD 2017 variaram entre 248,8 e 178,0 óbitos por 100 mil habitantes. É importante destacar que, de 2015 para 2017, observou-se, nas estimativas GBD, um aumento naquelas taxas, também observado no SIM Corrigido de 2015 a 2016.

**Figura 5:**
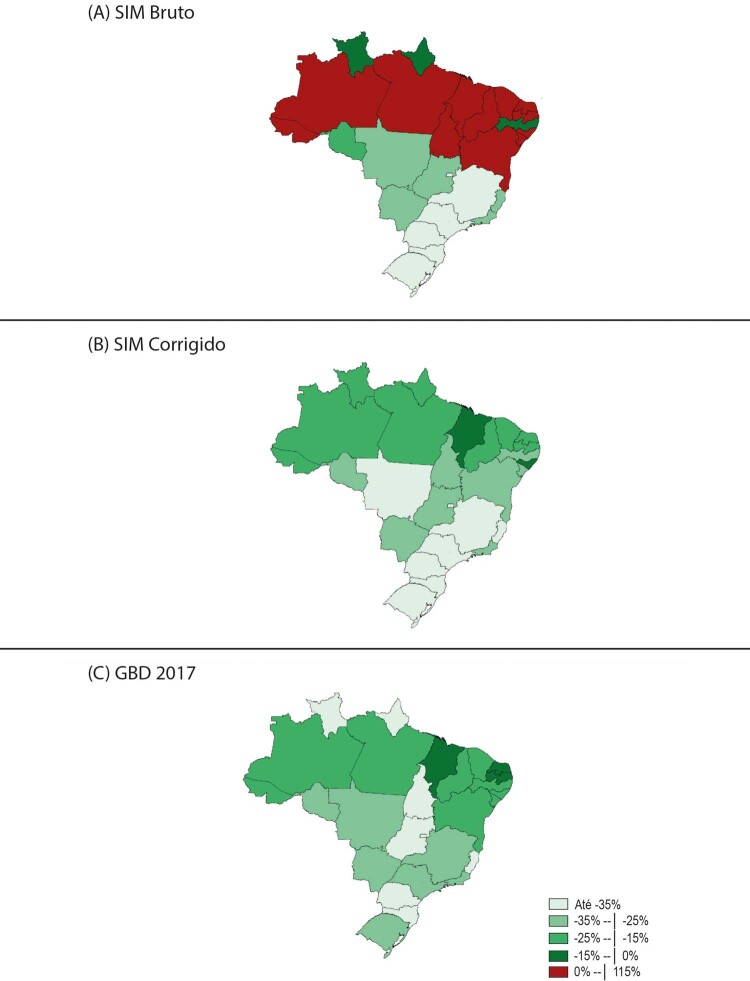
– *Variação porcentual entre 2000 e 2017 das taxas de mortalidade padronizadas por doenças cardiovasculares. Fonte: SIM e GBD 2017*

Ao analisar as variações porcentuais entre as taxas de mortalidade padronizadas por DCV entre os anos de 2000 e 2017 por UF, observou-se uma diferença que se destaca nos dados brutos do SIM, com estabilização das taxas ou aumento de até 115% na maioria dos estados das regiões Norte e Nordeste do país. Esse padrão não ocorre no SIM Corrigido nem no GBD, que, ao contrário, apresentaram redução nas taxas em todas as UF ( Figura 5 , Tabela 2 ).

**Tabela 2:** – Taxas de mortalidade padronizadas por doenças cardiovasculares para o Brasil e suas unidades da federação nos anos 2000 e 2017, assim como suas variações porcentuais no período.

Local	SIM Bruto			SIM Corrigido			GBD 2017		

	2000	2017	Variação (%)	2000	2017	Variação (%)	2000	2017	Variação (%)
Acre	122,4	155,4	26,9	199,1	167,6	-15,8	203,9	158,5	-22,3
Alagoas	159,5	210,7	32,1	261,7	225,7	-13,8	253,6	211,9	-16,4
Amapá	149,7	136,1	-9,0	200,9	160,8	-20,0	242,7	157,2	-35,2
Amazonas	124,2	128,6	3,5	188,4	156,0	-17,2	177,5	147,0	-17,1
Bahia	134,8	137,6	2,1	235,6	173,8	-26,2	210,0	162,9	-22,4
Ceará	139,6	158,5	13,6	219,7	165,6	-24,6	194,1	152,4	-21,5
Distrito Federal	233,1	135,6	-41,8	251,2	142,1	-43,4	301,5	175,4	-41,8
Espírito Santo	220,4	156,0	-29,2	278,6	158,6	-43,1	275,8	165,8	-39,9
Goiás	215,5	161,4	-25,1	247,3	166,6	-32,6	252,3	163,9	-35,1
Maranhão	83,5	179,3	114,8	225,5	211,1	-6,4	190,3	184,6	-3,0
Mato Grosso	228,4	152,1	-33,4	258,1	163,7	-36,6	240,2	162,8	-32,2
Mato Grosso do Sul	240,1	165,1	-31,2	270,5	177,0	-34,6	274,6	198,6	-27,7
Minas Gerais	204,6	132,9	-35,1	242,6	146,3	-39,7	228,4	154,5	-32,4
Pará	132,1	156,0	18,1	223,9	182,4	-18,5	200,9	168,6	-16,1
Paraíba	98,2	168,7	71,7	233,3	188,3	-19,3	213,0	190,9	-10,4
Paraná	287,4	152,8	-46,8	306,6	167,8	-45,3	297,0	188,3	-36,6
Pernambuco	206,0	183,2	-11,1	282,0	205,6	-27,1	263,2	214,6	-18,5
Piauí	136,5	190,6	39,6	269,0	201,9	-24,9	227,8	175,1	-23,1
Rio de Janeiro	252,1	168,1	-33,3	292,7	194,9	-33,4	296,0	207,7	-29,8
Rio Grande do Norte	121,9	145,7	19,6	196,6	156,0	-20,7	185,9	159,2	-14,3
Rio Grande do Sul	263,4	138,6	-47,4	277,1	154,6	-44,2	266,2	177,2	-33,4
Rondônia	191,7	157,3	-17,9	251,2	180,3	-28,2	253,0	184,8	-26,9
Roraima	190,5	177,5	-6,8	221,3	187,6	-15,2	305,9	196,3	-35,8
Santa Catarina	237,3	138,6	-41,6	279,9	150,0	-46,4	277,6	170,2	-38,7
São Paulo	264,3	160,3	-39,3	285,6	172,7	-39,5	283,3	185,6	-34,5
Sergipe	141,7	154,0	8,7	234,8	170,2	-27,5	218,6	171,6	-21,5
Tocantins	155,5	186,2	19,8	259,4	193,2	-25,5	294,6	173,9	-41,0

Brasil	211,7	155,1	-26,7	263,9	172,0	-34,8	248,8	178,0	-28,5

## Discussão

O estudo compara três diferentes métodos para estimar as séries históricas de mortalidade por DCV no Brasil entre 2000 e 2017. Em todo o período, ocorreu declínio das taxas de mortalidade por DCV, exceto após 2015, quando houve aumento nas estimativas das taxas no GBD e estabilidade delas no SIM Corrigido. As estimativas do SIM Corrigido e do GBD mostram-se semelhantes, em especial após 2006, quando houve melhora na qualidade do SIM. As taxas brutas, não corrigidas, apresentaram aumento para estados das regiões Norte e Nordeste, enquanto, nos demais métodos, todas as UF apontaram redução no período.

As DCV constituem a primeira causa de morte no mundo^13^ e no Brasil,^5 , 14^ correspondendo a um terço do total de óbitos. Todas as regiões apresentaram declínio da mortalidade por doenças crônicas não transmissíveis (DCNT). As DCV e suas complicações têm impacto elevado na perda de produtividade no trabalho e na redução da renda familiar, resultando em um déficit de US$ 4,18 bilhões na economia brasileira entre 2006 e 2015.^15^ Estudos realizados em vários países têm apontado redução na incidência e na mortalidade por DCV desde a década de 1960.^16 , 17^ No Brasil, esse declínio ocorreu mais tardiamente, na década de 1990.^5 , 14^

Ao longo dos anos, o SIM foi sendo aprimorado com vistas ao aumento da cobertura e à melhoria da qualidade do registro das causas básicas de morte na DO. Esses avanços resultaram de esforços do Ministério da Saúde em parcerias com as UF e os municípios para melhorar a captação dos óbitos pelo SIM, como o Projeto de Redução das Causas Mal Definidas no ano de 2005 e os Projeto de Redução das Desigualdades Regionais e de Redução da Mortalidade Infantil nos estados da região Nordeste e Amazônia Legal.^18^ Destaca-se ainda o Projeto de Busca Ativa do Óbito, que possibilitou definir metodologias para redistribuição de óbitos sub-registrados.^19 , 20^ Essas correções são essenciais para a correta interpretação e a comparabilidade das séries históricas nas diferentes regiões do país.

Destaca-se a redução importante do porcentual de causas mal definidas do SIM, refletindo a melhoria da qualidade dos serviços de assistência à saúde e o aumento da cobertura assistencial, em especial a expansão das equipes de saúde da família no interior do país.^5^

As diferenças entre o SIM corrigido e o GBD 2017 são também explicadas pelo porcentual de códigos de causas pouco úteis ou causas inespecíficas, também denominados na literatura internacional de CG.^8^ Enquanto as taxas apresentadas pelo SIM Corrigido não foram corrigidas pela redistribuição dos CG, ao contrário, o GBD utilizou em todas as estimativas a correção do sub-registro e a redistribuição de causas mal definidas e de CG ou causas inespecíficas. Assim, as estimativas do GBD apresentam taxas diferentes daquelas dos demais métodos que não usam a redistribuição dos CG. São exemplos de CG causas como septicemia, parada cardíaca, desidratação, insuficiência cardíaca congestiva, que fazem parte da cadeia de eventos que levaram ao óbito, mas não consistem na causa básica de morte.^8^ Para a redistribuição dos CG, o GBD utiliza algoritmos que se baseiam em evidências da literatura médica, múltiplas fontes, opiniões de especialistas, análise de causas múltiplas e, principalmente, em técnicas de modelagem estatística para definir o peso de designação de cada CG para as causas básicas mais prováveis de morte, chamadas de *target.* ^6 , 8^

O SIM é um sistema consolidado, para o qual o Ministério da Saúde vem buscando aperfeiçoamento ao longo dos anos, como, por exemplo, processos de validação de inconsistências internas e melhoria na notificação de óbitos. Entretanto, há que se avançar no refinamento da classificação, em especial na redução dos CG. Ao se comparar as estimativas de mortalidade do GBD com os dados do SIM Bruto, as diferenças observadas devem-se às inconsistências de idade e sexo relacionadas a causas de óbito, sub-registro e redistribuição dos CG *.* Essa última consiste na etapa que mais tem influência na alteração das estimativas corrigidas em relação às brutas. Os CG de nível 1 e 2, aqueles com pouca especificação da real causa de morte, correspondem a cerca de 12% dos registros do SIM. Quando se consideram os níveis 3 e 4 a serem redistribuídos dentro do mesmo grupo de causas, ou seja, com melhor especificação da causa de morte, os CG podem somar até 40%, o que pode resultar em diferenças nas estimativas entre SIM e GBD.^6 , 8^

O estudo aponta que as análises do SIM Bruto têm vieses, principalmente para estados das regiões Norte e Nordeste, não sendo recomendada sua adoção, em especial para a definição de políticas regionais, pois as taxas estão sujeitas a erros de estimativas, como sub-registro e alta proporção de causas mal definidas. São necessários ajustes metodológicos para cobertura e redistribuição de causas mal definidas, ainda mais em se tratando de análises das séries históricas em época em que a qualidade do SIM era mais comprometida.

Em 2015, a Assembleia das Nações Unidas aprovou os 17 Objetivos de Desenvolvimento Sustentável, dentre os quais figuram assegurar uma vida saudável e promover o bem-estar para todos, em todas as idades. Foi incluído o indicador “redução da probabilidade de morte prematura por DCNT em 30% até 2030”, que envolve no seu cálculo a redução das DCV. Cumprir as metas de redução de DCNT e DCV é um desafio global.^21 - 23^

Para atingir as metas de redução de DCNT, a Organização Mundial da Saúde divulgou um conjunto de evidências que aponta a importância das ações de promoção à saúde, implementando políticas públicas intra- e intersetoriais que facilitem práticas saudáveis, como alimentação adequada, redução do sal nos alimentos, disponibilização de espaços públicos para apoiar a atividade física, ambientes livres de fumo, regulamentação da propaganda de álcool e outras.^24^ Além disso, cabe o investimento na atenção básica e no acesso às tecnologias de média e alta complexidade, quando necessário, visando ao cuidado integral dos portadores de DCNT.^25 23^

O estudo aponta que, nos anos posteriores a 2015, ocorreu aumento das taxas de mortalidade por DCV (GBD 2017) ou sua estabilidade (SIM Corrigido). Esses dados devem ser revistos frente ao pequeno número de anos analisados. Entretanto, outros estudos já apontaram a piora dos indicadores em saúde no país, o que tem sido atribuído à crise econômica, ao aumento da pobreza, aos cortes em saúde e políticas sociais produzidos pela Emenda Constitucional n^o^95 e pelo congelamento dos recursos da saúde por 20 anos.^22 - 25^

Dentre as limitações do estudo, o uso de bases secundárias pode agregar vieses como o sub-registro e as inconsistências no preenchimento das causas de morte. As estimativas populacionais no país também podem estar sujeitas a erros, dado que o último censo no Brasil data de 2010. As estimativas do GBD também podem apresentar limitações devido às suas fontes, aos ajustes e aos algoritmos empregados.

## Conclusão

Este estudo aponta a diminuição das taxas de mortalidade por DCV no período analisado, exceto nos dois últimos anos. As estimativas comparadas apontam semelhanças entre o SIM Corrigido e o GBD 2017. Não se recomenda o uso dos dados brutos do SIM, em especial para análises subnacionais, pois pode resultar em erros na interpretação, com o aumento das taxas podendo decorrer não apenas do aumento na captação de óbitos como também da melhoria na definição das causas básicas de morte na última década, em especial nas regiões Norte e Nordeste. Isso justifica que sempre se utilizem dados corrigidos na análise de mortalidade.
