# Supplementary material for: Mortalidade por Doenças Cardiovasculares Segundo o Sistema de Informação sobre Mortalidade e as Estimativas do Estudo Carga Global de Doenças no Brasil, 2000-2017
Source: Arq Bras Cardiol. 2020 Aug 19;115(2):152–60. [Article in Portuguese] doi: 10.36660/abc.20190867 (PMC8384283; doi:10.36660/abc.20190867)
Supplement: Translation

# Translation: Cardiovascular Disease Mortality According to the Brazilian Information System on Mortality and the Global Burden of Disease Study Estimates in Brazil, 2000-2017

**Authors:** Deborah Carvalho Malta, Renato Teixeira, Gláucia Maria Moraes de Oliveira, Antonio Luiz Pinho Ribeiro

**Affiliations:** 1 Brazil Universidade Federal de Minas Gerais - Escola de Enfermagem, Programa de Pós-Graduação em Saúde Pública., Belo Horizonte, MG - Brazil; 2 Brazil Universidade Federal de Minas Gerais Programa de Pós-Graduação em Saúde Pública, Faculdade de Medicina, Belo Horizonte, MG - Brazil; 3 Brazil Universidade Federal do Rio de Janeiro, Rio de Janeiro, RJ - Brazil; 4 Brazil Universidade Federal de Minas Gerais - Centro de Telessaúde - Hospital das Clínicas/Departamento de Clínica Médica - Faculdade de Medicina, UFMG, Belo Horizonte, MG – Brazil

**Keywords:** Cardiovascular Diseases/mortality, Data Accuracy/trends, Health Information System/trends, Epidemiology

## Abstract

**Background:**

The Brazilian Information System on Mortality (SIM) is of vital importance in monitoring the trends of cardiovascular diseases (CVDs) and is aimed at supporting public policies.

**Objective:**

To compare historical series of CVD mortality based on data from the SIM, with and without correction, and from the Brazil Global Burden of Disease (GBD) Study 2017, in the 2000-2017 period.

**Methods:**

Analysis of CVD mortality in Brazil between 2000 and 2017. Three CVD mortality estimates were compared: Crude SIM, Corrected SIM, and GBD 2017. Absolute numbers and age-standardized rates were used to compare the estimates for Brazil, its states and the Federal District.

**Results:**

In the SIM, the total of deaths ranged from 261,000, in 2000, to 359,000, in 2017. In the GBD 2017, the total of deaths ranged from 292,000 to 388,000, for the same years, respectively. A high proportion of the causes of death from CVD corresponded to garbage codes, classified according to the GBD 2017, reaching 42% in 2017. The rates estimated by GBD ranged from 248.8 (1990) to 178.0 (2017) deaths per 100,000 inhabitants. The rates of the Crude SIM and Corrected SIM also showed a reduction for the whole series analyzed, the Crude SIM showing lower rates: 204.9 (1990) and 155.1 (2017) deaths per 100 thousand inhabitants. When analyzing by the states and Federal District, the Crude SIM trends reversed, with an increase in mortality rates in the Northern and Northeastern states.

**Conclusion:**

This study shows the decrease in CVD mortality rates in Brazil in the period analyzed. Conversely, when analyzing by the states and Federal District, the Crude SIM showed an increase in those rates for the Northern and Northeastern states. The use of crude data from the SIM can result in interpretation errors, indicating an increase in rates, due to the increase in death data capture and the improvement in the definition of the underlying causes of death in the past decade, especially in the Northern and Northeastern regions, justifying the use of corrected data in mortality analyses. (Arq Bras Cardiol. 2020; 115(2):152-160)

## Introduction

In past years, Brazil has compiled different data sources that constitute the information system on morbidity and mortality and periodic health inquiries, which enable the continuous monitoring of data on mortality, morbidity, and risk factors for cardiovascular diseases (CVDs), and support decision-making processes in health policies.^1 , 2^

The Brazilian Information System on Mortality (SIM), which provides data to identify and address death record information, was implanted in 1975, being the first nationwide epidemiological database of the Brazilian Health Ministry.^3^ The cornerstone of SIM is the death certificate, which should be completed by the physician caring for the deceased patient. In the absence of that professional, however, the death certificate can be completed by: the substitute physician; the physician from the Death Verification Service – for natural causes of death; or the coroner – for deaths by external causes.^2 , 3^

All Brazilian municipalities must register their deaths, which results in around 1.3 million deaths reported per year, making SIM one of the major tools to monitor the mortality statistics in Brazil. The SIM coverage has increased in all Brazilian states and Federal District, passing from 86% in 2000 to 98% in 2017; however, some Northern and Northeastern states maintain coverages lower than 95%.^1 - 3^ In addition, the number of ill-defined causes of death in Brazil has decreased, although it still remains high in some states. Therefore, the analyses of health status based on mortality records should be carried out with corrective methodologies that can minimize the bias caused by ill-defined causes of death, garbage codes (GC), and death underreporting.^4 - 6^

Since 1990, the Global Burden of Disease (GBD) Study has adopted a methodology that consists in large advances and in a paradigm change in the epidemiological analysis of secondary data, by proposing an integrated focus on diseases and deaths, with robust and standardized methodology of analysis that contemplates the correction of GC, ill-defined causes of death, and death underreporting.^7^ The GBD Study provides comprehensive information on 249 causes of death in 195 locations, contemplating countries and some subnational levels, such as Brazil and its 26 states and Federal District. In the GBD Study, the information on causes of death has been collected from vital record systems, mortality surveillance systems, research, hospital records, police records, and verbal autopsies. For Brazil and its 26 states and Federal District, the SIM is the data source on mortality.^7 , 8^ In the GBD Study, several statistical models are used to best estimate the number of deaths per each cause of death according to sex and age. The GBD Study enables comparisons between countries, regions, and subnational data, because the quality of the local mortality data is standardized. In addition, the GBD Study enables the analysis of population trends, because the temporal series data are corrected and standardized, making comparison over time possible.^7 - 10^

This study was aimed at comparing historical series of CVD mortality based on data from the SIM, with and without correction, and the Brazil GBD Study 2017 estimates.

## Methods

This study assessed the historical series of CVD mortality in Brazil from 2000 to 2017. The data source for this study was the SIM, which contains the major information on death records in the whole country. Initially, the proportions of ill-defined causes of death in the SIM were described ( Figure 1 ).

**Figure 1:** – *Proportion of ill-defined causes of death in the Brazilian Information System on Mortality (SIM), Brazil, 2000 - 2017. Source: SIM.*

Three estimates of CVD mortality were compared: Crude SIM, Corrected SIM, and Brazil GBD Study 2017. The estimates deriving from the SIM, with and without correction, named Corrected SIM and Crude SIM, respectively, used the definition of CVDs in accordance with the 10^th^Revision of the International Statistical Classification of Diseases and Related Health Problems (ICD-10) chapter IX codes (diseases of the circulatory system - I00-I99). The GBD classification considered initially the following codes: B33.2, G45-G46.8, I01-I01.9, I02.0, I05-I09.9, I11-I11.9, I20-I25.9, I28-I28.8, I30-I31.1, I31.8-I37.8, I38-I41.9, I42.1-I42.8, I43-I43.9, I47-I48.9, I51.0-I51.4, I60-I63.9, I65-I66.9, I67.0-I67.3, I67.5-I67.6, I68.0-I68.2, I69.0-I69.3, I70.2-I70.8, I71-I73.9, I77-I83.9, I86-I89.0, I89.9, I98, and K75.1.

Figure 2 shows the methods for correcting death and population data used to estimate the absolute numbers and mortality rates for the Crude SIM and Corrected SIM, as well as the GBD Study 2017 estimates. The numerator corresponds to the CVDs (I00-I99) registered by the SIM. The estimates generated by the Crude SIM were age-standardized, no other correction being applied. The estimates generated by the Corrected SIM were age-standardized and corrected as follows: for underreporting, by using the GBD methodology; for the deaths lacking information on age and sex, by using the proportional redistribution of these deaths; and for the ill-defined causes of death, by using the proportional redistribution of these causes in the groups of cardiovascular causes and the other ICD-10 chapters.^4^ The GBD 2017 estimates were extracted from the Institute for Health Metrics and Evaluation (IHME) database, underwent the previously described corrections, such as for underreporting, GC, and ill-defined causes of death, and were detailed in previous publications.^7 , 8 , 10^

**Figure 2:** Flowchart of the estimates of the Crude and Corrected Information System on Mortality and the Global Burden of Diseases Study, methods for correction and population used, for absolute numbers and mortality rates. CVD: Cardiovascular Diseases; IBGE: Brazilian Institute of Geography and Statistics; SIM: Brazilian Information System on Mortality; GBD: Global Burden of Diseases Study; IDCD: Ill-Defined Causes of Death.

Data from the Crude SIM and Corrected SIM were compared with the estimates of the GBD Study 2017, which also uses SIM data as a source, both by using the 2000-2017 historical series of the total of deaths and absolute numbers of the diseases listed in the ICD-10 IX chapter *,* and the age-standardized rates of the three estimates. To calculate the rates in the Crude SIM and the Corrected SIM, the updated population estimates generated by the Brazilian Institute of Geography and Statistics (IBGE) were used as denominator.^11^ However, that IBGE estimate only provides data from 2010 onwards; thus, two interpolations were applied: one with data from the year 2000 of the 2013 version made available by IBGE,^12^ and another interpolation with data from the year 2010 of the current version made available in 2018.^11^ The standard population used to adjust the age-standardized rates, through the direct method, was the world population of the GBD Study.^7^ To calculate the rates, the GBD Study considers its own population estimates (Source GBD). All three estimates were analyzed for the ICD-10 chapter IX codes (diseases of the circulatory system - I00-I99) from 2000 to 2017. Figure 2 shows the flowchart used to compare the three estimates, regarding the absolute numbers of deaths and mortality rates for Brazil, its states, and Federal District.

The analyses were performed using Stata Statistical Software: Release 14. College Station, TX: StataCorp LP.

## Results

Figure 1 shows the proportion of ill-defined causes of death in relation to the total number of deaths in Brazil from 2000 to 2017. That proportion was 14.3% in 2000 and decreased over time, more abruptly from 2005 onward, reaching 5.5% in 2017.

Table 1 shows that a large number of deaths with underlying causes classified as ICD-10 chapter IX codes constituted GC, as by the definition of GC in the GBD Study 2017, and that proportion was 42.1% in 2017. In addition, Table 1 shows that the number of GC decreased slowly over the years, indicating an improvement in the quality of the definition of the ICD-10 chapter IX causes of death. The GBD Study redistributes the GC in its estimates.

**Table 1:** – Total of deaths, absolute numbers and percentages of deaths due to cardiovascular diseases according to the ICD-10 IX chapter codes (I00-I99) and to the definitions of GBD for cardiovascular diseases, and absolute numbers and percentages of garbage codes, in Brazil, 2000 to 2017

Year	Total	I00-I99		Cardiovascular GBD		Garbage codes	

		n	%	n	%	n	%
2000	946,685	260,603	27.5	129,883	49.8	126,803	48.7
2001	961,492	263,417	27.4	130,774	49.6	128,637	48.8
2002	982,807	267,496	27.2	133,160	49.8	130,362	48.7
2003	1,002,340	274,068	27.3	137,413	50.1	132,245	48.3
2004	1,024,073	285,543	27.9	143,811	50.4	137,096	48.0
2005	1,006,827	283,927	28.2	142,656	50.2	136,545	48.1
2006	1,031,691	302,817	29.4	152,017	50.2	145,977	48.2
2007	1,047,824	308,466	29.4	156,253	50.7	147,076	47.7
2008	1,077,007	317,797	29.5	163,255	51.4	149,058	46.9
2009	1,103,088	320,074	29.0	164,036	51.2	150,082	46.9
2010	1,136,947	326,371	28.7	167,974	51.5	152,326	46.7
2011	1,170,498	335,213	28.6	173,397	51.7	155,363	46.3
2012	1,181,166	333,295	28.2	174,750	52.4	152,276	45.7
2013	1,210,474	339,672	28.1	179,200	52.8	153,822	45.3
2014	1,227,039	340,284	27.7	181,223	53.3	152,421	44.8
2015	1,264,175	349,642	27.7	186,570	53.4	156,278	44.7
2016	1,309,774	362,091	27.6	194,987	53.9	159,779	44.1
2017	1,312,664	358,882	27.3	199,872	55.7	150,967	42.1

*Deaths of the cardiovascular chapter not classified as cardiovascular diseases, and garbage codes not presented in the table, mean of 1.8%.

The absolute numbers of deaths due to CVD and standardized mortality rates of the Crude SIM, the Corrected SIM, and the GBD Study 2017 estimates were analyzed. Figure 3 depicts the absolute number of deaths due to CVD for the three estimates, with a similar increase for all three methods. The SIM registered approximately 261,000 deaths in 2000, reaching 359,000 deaths in 2017. After data correction, the SIM records ranged from 324,000 in 2000 to 397,000 in 2017. The GBD 2017 estimates increased from 292,000 deaths to 388,000 deaths in those same years.

**Figure 3:** – *Absolute numbers of cardiovascular disease deaths according to the Crude and Corrected SIM and the GBD Study 2017. Brazil, 2000 to 2017. Sources: SIM and Brazil GBD Study 2017.*

The CVD mortality rates decreased in the period analyzed ( Figure 4 ). The Crude SIM rates decreased from 211.7 to 155.1 deaths per 100,000 inhabitants, while the Corrected SIM rates decreased from 263.9 to 172.0 deaths per 100,000 inhabitants. The GBD 2017 estimated rates decreased from 248.8 to 178,0 deaths per 100,000 inhabitants. However, it is worth noting that, from 2015 to 2017, the GBD estimated rates increased, and this increase was also observed in the Corrected SIM rates from 2015 to 2016.

**Figure 4:** – *Standardized cardiovascular disease mortality rates according to the Crude and Corrected SIM and the GBD Study 2017. Brazil, 2000 to 2017. Sources: SIM and Brazil GBD Study 2017.*

When assessing the percentage variations in the standardized CVD mortality rates from 2000 to 2017 by each state and the Federal District, there was a difference in the Crude SIM data, with stabilization in the rates or their increase (of as much as 115%) in most Northern and Northeastern states. That pattern is observed neither in the Corrected SIM data nor in the GBD Study data, whose rates showed a reduction in all Brazilian states and the Federal District ( Figure 5 , Table 2 ).

**Figure 5:** – *Percentage variation of standardized cardiovascular mortality rates from 2000 to 2017. Sources: SIM and Brazil GBD Study 2017.*

**Table 2:** – Standardized cardiovascular disease mortality rates for Brazil, its states and Federal District, in the years 2000 and 2017, as well as their percentage variations in the period

Location	Crude SIM			Corrected SIM			GBD 2017		

	2000	2017	Variation (%)	2000	2017	Variation (%)	2000	2017	Variation (%)
Acre	122.4	155.4	26.9	199.1	167.6	-15.8	203.9	158.5	-22.3
Alagoas	159.5	210.7	32.1	261.7	225.7	-13.8	253.6	211.9	-16.4
Amapá	149.7	136.1	-9.0	200.9	160.8	-20.0	242.7	157.2	-35.2
Amazonas	124.2	128.6	3.5	188.4	156.0	-17.2	177.5	147.0	-17.1
Bahia	134.8	137.6	2.1	235.6	173.8	-26.2	210.0	162.9	-22.4
Ceará	139.6	158.5	13.6	219.7	165.6	-24.6	194.1	152.4	-21.5
Federal District	233.1	135.6	-41.8	251.2	142.1	-43.4	301.5	175.4	-41.8
Espírito Santo	220.4	156.0	-29.2	278.6	158.6	-43.1	275.8	165.8	-39.9
Goiás	215.5	161.4	-25.1	247.3	166.6	-32.6	252.3	163.9	-35.1
Maranhão	83.5	179.3	114.8	225.5	211.1	-6.4	190.3	184.6	-3.0
Mato Grosso	228.4	152.1	-33.4	258.1	163.7	-36.6	240.2	162.8	-32.2
Mato Grosso do Sul	240.1	165.1	-31.2	270.5	177.0	-34.6	274.6	198.6	-27.7
Minas Gerais	204.6	132.9	-35.1	242.6	146.3	-39.7	228.4	154.5	-32.4
Pará	132.1	156.0	18.1	223.9	182.4	-18.5	200.9	168.6	-16.1
Paraíba	98.2	168.7	71.7	233.3	188.3	-19.3	213.0	190.9	-10.4
Paraná	287.4	152.8	-46.8	306.6	167.8	-45.3	297.0	188.3	-36.6
Pernambuco	206.0	183.2	-11.1	282.0	205.6	-27.1	263.2	214.6	-18.5
Piauí	136.5	190.6	39.6	269.0	201.9	-24.9	227.8	175.1	-23.1
Rio de Janeiro	252.1	168.1	-33.3	292.7	194.9	-33.4	296.0	207.7	-29.8
Rio Grande do Norte	121.9	145.7	19.6	196.6	156.0	-20.7	185.9	159.2	-14.3
Rio Grande do Sul	263.4	138.6	-47.4	277.1	154.6	-44.2	266.2	177.2	-33.4
Rondônia	191.7	157.3	-17.9	251.2	180.3	-28.2	253.0	184.8	-26.9
Roraima	190.5	177.5	-6.8	221.3	187.6	-15.2	305.9	196.3	-35.8
Santa Catarina	237.3	138.6	-41.6	279.9	150.0	-46.4	277.6	170.2	-38.7
São Paulo	264.3	160.3	-39.3	285.6	172.7	-39.5	283.3	185.6	-34.5
Sergipe	141.7	154.0	8.7	234.8	170.2	-27.5	218.6	171.6	-21.5
Tocantins	155.5	186.2	19.8	259.4	193.2	-25.5	294.6	173.9	-41.0

Brazil	211.7	155.1	-26.7	263.9	172.0	-34.8	248.8	178.0	-28.5

## Discussion

This study compares three different methods to estimate the historical series of CVD mortality in Brazil from 2000 to 2017, which decreased, except for the period from 2015 onwards, when there was an increase in the GBD estimated rates and stability in the Corrected SIM rates. The estimates of the Corrected SIM and GBD were similar, especially after 2006, when the quality of the SIM improved. The Crude SIM showed an increase in the rates of the Northern and Northeastern states, while the Corrected SIM and GBD showed a reduction in the rates of all states and the Federal District in the period.

Cardiovascular diseases are the number one cause of death globally^13^ and in Brazil,^5 , 14^ corresponding to one third of all deaths. All regions showed a decline in mortality due to chronic non communicable diseases (CNCDs). The CVDs and their complications have a high impact on the loss of productivity in the workplace and in the family income, resulting in a US$ 4.18 billion deficit in the Brazilian economy from 2006 to 2015.^15^ Studies conducted in several countries have shown a reduction in the incidence of CVDs and in CVD mortality since the 1960s.^16 , 17^ In Brazil, that reduction occurred later, in the 1990s.^5 , 14^

Over the years, SIM has been improved, having its coverage increased and the quality of the reports of underlying causes of death in death certificates refined. These advances have resulted from the efforts of the Brazilian Ministry of Health in partnership with states and municipalities to improve death data collection by the SIM. Some examples are the 2005 Project to Reduce Ill-Defined Causes and the Projects to Reduce Regional Inequalities and Child Mortality in the Northeastern states and the ‘Legal Amazonia’ region.^18^ It is worth noting the Project of Active Search for Death Data, which enabled the definition of methodologies to redistribute underreported deaths.^19 , 20^ Those corrections are essential for the accurate interpretation and comparability of historical series in different regions of Brazil.

It is worth noting the important reduction in the percentage of ill-defined causes of death in the SIM, which results from the improvement in the quality of healthcare services and the increase in healthcare coverage, especially the advance of the family healthcare team to inner areas of the country.^5^

In addition, the differences between the Corrected SIM and the GBD Study 2017 can be explained by the percentage of the codes of unspecific causes, named ‘garbage code’ in the international literature.^8^ Although the rates provided by the Corrected SIM were not corrected by the redistribution of GC, all GBD Study estimates were corrected for death underreporting and redistribution of ill-defined causes and of GC. Thus, the GBD estimated rates differ from those of the other methods that do not use GC redistribution. Some examples of GC causes are as follows: septicemia; cardiac arrest; dehydration; congestive heart failure. They are part of the train of events leading to death, but are not the underlying cause of death.^8^ For the GC redistribution, the GBD Study uses algorithms based on medical literature evidence, multiple sources, expert opinions, analysis of multiple causes, and mainly on statistical modelling techniques to define the weight of assigning each GC to the most probable underlying cause of death, called target *.* ^6 , 8^

The SIM is a consolidated system, which the Brazilian Ministry of Health has been perfecting via processes of validation of internal inconsistencies and improvement in death reporting. However, the classification requires refinement, especially regarding the reduction in GC. When comparing the mortality estimates of the GBD Study with the Crude SIM data, the differences observed are due to age and sex inconsistencies related to causes of death, underreporting, and GC redistribution *.* The GC redistribution has the greatest influence on the difference between corrected estimates and crude ones. Level 1 and level 2 GC, those with little specification of the real cause of death, correspond to 12% of the SIM records. When level 3 and level 4 GC are considered to be redistributed in the same group of causes, that is, with better specification of the cause of death, the GC can reach 40%, which can lead to differences in the estimates between the SIM and the GBD.^6 , 8^

This study indicates that the Crude SIM analyses are biased, especially for the Northern and Northeastern states. Therefore, its adoption is not recommended, especially for the definition of regional policies, because the rates are subject to estimation errors, such as those due to underreporting and excessive proportion of ill-defined causes. Methodological adjustments to coverage and redistribution of ill-defined causes are necessary, even more when it comes to the analysis of historical series, involving a period in which the quality of SIM was impaired.

In 2015, the United Nations Assembly approved the Sustainable Development Goals, representing 17 global challenges to achieve a better and more sustainable future for all. One of the goals is to ensure good health and well-being for all, at all ages. It includes the indicator “a 30% reduction in premature mortality from CNCDs by 2030”, whose calculation involves a reduction in CVDs. Reducing CNCDs and CVDs is a global challenge.^21 - 23^

To meet the goal to prevent and control CNCDs, the World Health Organization has issued a document recommending the adoption of interventions for health promotion, with the implementation of public policies within and across sectors that promote healthy practices, such as healthy diets, low-sodium food products, open public spaces and adequate infrastructure to support physical activity, smoke-free environments, and alcohol advertising regulation.^24^ In addition, it is worth noting the importance of investing in primary care and access to medium and high complexity technologies, when necessary, aimed at the whole care of patients with CNCDs.^25^

This study shows an increase (GBD 2017) or stability (Corrected SIM) in the mortality rates due the CVD from 2015 onwards. These data need to be revised because of the short period analyzed. However, other studies have reported worsening of health indicators in Brazil, which has been attributed to the economic crisis, the increase in poverty, and the cuts in health and social policies resulting from the Constitutional Amendment 95/2016 and the freeze on public expenses, health included, for 20 years.^22 - 25^

This study has limitations. The use of secondary databases can add biases, such as underreporting and inconsistencies in death certificate completion. In addition, the Brazilian population estimates may be subject to errors, as the last available census in Brazil dates back to 2010. Moreover, the GBD estimates might have limitations because of its sources, adjustments, and algorithms used.

## Conclusion

This study shows the decline in the CVD mortality rates in the period analyzed, except for the last two years. The comparison of the estimates shows similarities between the Corrected SIM and the GBD Study 2017. However, the use of the Crude SIM data is not recommended, especially for subnational analyses, because it can result in interpretation errors. In this study, the increase in mortality rates might have reflected the improvement in death data capture and in the definition of the underlying causes of death in the past decade, especially in the Northern and Northeastern regions. This justifies the recommendation to always use corrected data for mortality analyses.
